# Role of IQGAP1 in Carcinogenesis

**DOI:** 10.3390/cancers13163940

**Published:** 2021-08-04

**Authors:** Tao Wei, Paul F. Lambert

**Affiliations:** McArdle Laboratory for Cancer Research, Department of Oncology, University of Wisconsin School of Medicine and Public Health, Madison, WI 53705, USA; twei27@wisc.edu

**Keywords:** PI3K, Ras, Wnt, scaffold, cancer, HPV

## Abstract

**Simple Summary:**

IQ motif-containing GTPase-activating protein 1 (IQGAP1) is a signal scaffolding protein that regulates a range of cellular activities by facilitating signal transduction in cells. IQGAP1 is involved in many cancer-related activities, such as proliferation, apoptosis, migration, invasion and metastases. In this article, we review the different pathways regulated by IQGAP1 during cancer development, and the role of IQGAP1 in different types of cancer, including cancers of the head and neck, breast, pancreas, liver, colorectal, stomach, and ovary. We also discuss IQGAP1′s regulation of the immune system, which is of importance to cancer progression. This review highlights the significant roles of IQGAP1 in cancer and provides a rationale for pursuing IQGAP1 as a drug target for developing novel cancer therapies.

**Abstract:**

Scaffolding proteins can play important roles in cell signaling transduction. IQ motif-containing GTPase-activating protein 1 (IQGAP1) influences many cellular activities by scaffolding multiple key signaling pathways, including ones involved in carcinogenesis. Two decades of studies provide evidence that IQGAP1 plays an essential role in promoting cancer development. IQGAP1 is overexpressed in many types of cancer, and its overexpression in cancer is associated with lower survival of the cancer patient. Here, we provide a comprehensive review of the literature regarding the oncogenic roles of IQGAP1. We start by describing the major cancer-related signaling pathways scaffolded by IQGAP1 and their associated cellular activities. We then describe clinical and molecular evidence for the contribution of IQGAP1 in different types of cancers. In the end, we review recent evidence implicating IQGAP1 in tumor-related immune responses. Given the critical role of IQGAP1 in carcinoma development, anti-tumor therapies targeting IQGAP1 or its associated signaling pathways could be beneficial for patients with many types of cancer.

## 1. Introduction

IQ motif-containing GTPase-activating protein 1 (IQGAP1) belongs to the IQGAP protein family, which is evolutionarily conserved among eukaryotes [1,2]. IQGAP1 was first identified in 1994 as a novel sequence from human osteosarcoma tissue and was proposed to function as a GTPase-activating protein (GAP) that facilitates signal termination based upon its sequence similarity to those of other known GAPs [3]. However, later research showed that instead of turning off signals, IQGAP1 inhibits the intrinsic GTPase activities of binding partners such as RAC1 and CDC42, thereby stabilizing the active form of these G proteins [4]. IQGAP1 is a large protein with a size of 190 kDa (1657 amino acids (aa)) that is ubiquitously expressed [3]. This protein has five main domains through which it binds to other proteins: a calponin-homology domain (CHD, 44–159 aa), a poly-proline protein–protein domain (WW, 681–710 aa), a domain containing four IQ-motif (IQ, 745–864 aa), a Ras GAP-related domain (GRD, 1004–1237 aa), and a Ras GAP C-terminal domain (RGCT 1276–1657 aa) [5]. These binding domains mediate IQGAP1′s interaction with over 100 protein binding partners and, therefore, participate in various biological activities, including cytoskeletal dynamics, cell–cell adhesion, cell motility/invasion, and cell proliferation [1,5,6,7,8,9,10]. Acting as a scaffolding protein for multiple key oncogenic pathways, IQGAP1 has been investigated for years as an oncogene that promotes cancer [11,12]. In this review, we describe the different oncogenic pathways affected by IQGAP1, summarize the role of IQGAP1 in different types of cancer, and discuss the emerging evidence of IQGAP1 in regulating tumor immunology.

## 2. IQGAP1 Mediates Multiple Key Oncogenic Pathways

### 2.1. MAPK Signaling

MAPK signaling is one of the most critical signaling pathways in cancer development. This pathway transmits extracellular signals received by receptors, such as the epidermal growth factor receptor (EGFR), through a signaling cascade comprised of a series of kinases, i.e., the RAS-RAF-MEK-ERK cascade, that mediate transmission of signals from the cell surface to the nucleus and regulate cellular processes including cell growth, proliferation, apoptosis, differentiation, and migration [13]. IQGAP1 mediates the MAPK signaling pathway by binding to different components in the pathway, thereby serving as a scaffold [14,15,16,17]. First, IQGAP1 binds to EGFR and mediates its activation [14]. IQGAP1 also binds to Raf [15], MEK [17], and ERK [16,17,18] through its different binding domains. It is controversial whether IQGAP1 binds to RAS, with the latest evidence indicating no interaction between the two proteins [4,19,20]. Loss of IQGAP1 reduces the activation of these kinases and therefore results in less active MAPK signaling [14,15,16,17,18]. By facilitating MAPK signaling, IQGAP1 contributes to carcinogenesis by promoting cell proliferation, migration, invasion, angiogenesis, and metastasis [12,21,22].

IQGAP1 is also linked to the JNK pathway in the context of cell–cell adhesion [6,23]. The loss of IQGAP1 increases JNK activation through CDC42, a well-studied binding partner of IQGAP1, and promotes the formation of tight junctions [23]. The interaction between IQGAP1 and CDC42 will be discussed in the next section.

### 2.2. RAC1/CDC42

The Rho family of small GTPases or G proteins, RAC1 and CDC42, were the first binding partners identified for IQGAP1 [4,24]. IQGAP1 binds to and stabilizes GTP-bound, active RAC1 and CDC42 through its GRD domain [4,24]. IQGAP1 binds to F-actin through its CHD domain to mediate actin crosslinking, which is enhanced by the binding of active CDC42 and diminished by binding Ca^2+^/Calmodulin [25,26,27]. Post-translational modifications (PTMs) of IQGAP1 impact its interaction with CDC42: phosphorylation of IQGAP1 at Ser1443 increases its binding to CDC42, while the ubiquitination of IQGAP1 reduces its binding [28,29]. RAC1/CDC42 binding promotes other IQGAP1-mediated crosslinking between actin and microtubules by recruiting other effectors, such as CLIP-170, CLASP2, and APC, to interact with IQGAP1 [1,30,31,32]. By regulating cytoskeleton organization, IQGAP1 influences cell migration, invasion, adhesion, and metastasis during cancer development [8,12,33,34,35,36,37,38]. In this context, IQGAP1 often colocalizes with Rho GTPases at the leading edge of the invasive front of cancer cells [33,39,40].

### 2.3. Wnt Signaling

Wnt signaling is critical for tissue homeostasis, development, and fate decision and is often dysregulated in tumorigenesis [41]. IQGAP1 can bind to multiple proteins within this pathway, including Wnt receptors, LGR4 and LGR5 [35,42]; APC protein, which is one of the main regulators of Wnt signaling [32]; Dishevelled (Dvl), which mediates Wnt signaling into the cytoplasm [43]; and β-catenin, the ultimate intracellular mediator of Wnt signaling, in which IQGAP1 increases the level of nuclear-localized β-catenin [44]. Binding between IQGAP1 and β-catenin at adherent junctions can disrupt the α-β-catenin complex and decrease cell–cell adhesion [6]. Proposed models of IQGAP1 involvement in Wnt signaling have been described previously [1,10].

### 2.4. PI3K Signaling

PI3K signaling is often increased in many different types of cancer, driving cell proliferation, survival, and migration [45]. IQGAP1 scaffolds the different factors that make up the PI3K signaling pathway [46,47,48,49]. By binding to the series of kinases, PI4K, PIPKIα, and PI3K, IQGAP1 facilitates the formation of phosphatidylinositol-(3,4,5)-trisphosphate (PIP3) on the endosomal membrane upon receptor activation [48]. IQGAP1 also interacts with and modulates the activation of downstream effectors, including PDK1 and AKT [48]. The IQ3 motif on IQGAP1 is required for its interaction with PI3K-AKT signaling, which mediates IQGAP1-associated cell proliferation, survival, migration, and invasion in cancer [48,50,51].

### 2.5. Hippo Signaling

The Hippo pathway is an evolutionarily conserved pathway that controls organ growth [52]. Dysregulation in the Hippo pathway often presents in cancers [52]. IQGAP1 interacts with key components of the Hippo pathway, MST2 and LATS1, through its IQ domain and inhibits their activities [53]. The IQ domain of IQGAP1 also binds directly to the TEAD-binding domain of YAP1, a key effector of the Hippo pathway, and therefore competes with TEAD for YAP1 binding [53,54]. IQGAP1 thereby prevents YAP1-TEAD transcription [53,54]. Yet, YAP-TEAD interaction increases when IQGAP1 is overexpressed or knocked out [53,54]. Overexpression of IQGAP1 also interrupts the YAP1-p73 complex to prevent YAP1-associated pro-apoptotic signaling [53]. It is not clear whether IQGAP1 promotes the nuclear localization of YAP1. IQGAP1 does not promote YAP1 nuclear importation in mouse embryonic fibroblasts [54], but does so in a mouse model in the presence of overactive Wnt signaling and MET receptor kinase signaling [55]. Clearly, additional studies are needed to understand how IQGAP1 interacts and affects Hippo signaling.

### 2.6. TGF-β Signaling

TGF-β signaling has been long implicated in cancer progression, regulating multiple cellular processes, including cell proliferation, differentiation, apoptosis, and invasion, both in tumor cells and the tumor microenvironment [56]. Some studies have suggested a link between IQGAP1 and TGF-β signaling. IQGAP1 can interact with both type 1 and type 2 TGF-β receptors [57,58]. However, the interaction between IQGAP1 and TGF-β signaling differs depending on cell types. In liver pericytes, IQGAP1 binds to TGF-β receptor 2 and suppresses its downstream signaling, which inhibits TGF-β-induced differentiation [57]. In lung fibroblasts, TGF-β ligand binding inhibits IQGAP1 expression level via the NF-κB signaling pathway, which promotes fibroblast differentiation [59]. However, in mouse mammary gland epithelial cells, TGF-β upregulates IQGAP1 expression [60]. TGF-β-treated mammary gland epithelial cells show decreased growth and increased features of epithelial-mesenchymal transition (EMT), which may or may not be related to the IQGAP1 upregulation [60]. These discrepancies in the relationship between TGF-β and IQGAP1 may be due to tissue specificity or cell type specificity, or the complex roles that TGF-β signaling plays at different stages of cancer progression [56].

## 3. IQGAP1 Plays an Essential Role in Many Types of Cancer

**Figure 1:**
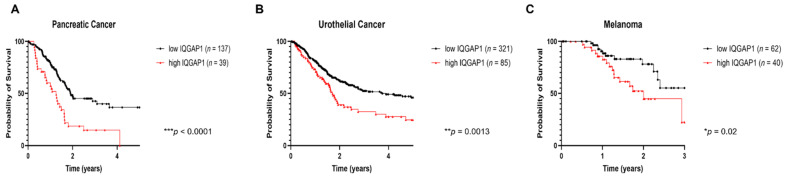
Summary of TCGA RNAseq data showing high levels of IQGAP1 is associated with poorer prognosis in cancers of pancreas, urethra, and melanoma. TCGA RNA-seq data were accessed through the Human Protein Atlas website (https://www.proteinatlas.org/ENSG00000140575-IQGAP1/pathology (accessed on 03 Aug 2021)). Cutoff for IQGAP1 levels was suggested by the database as shown on the website and is based upon on survival analysis that yields the maximal difference in survival between the two groups at the lowest log-rank *p*-value. Kaplan–Meier survival analysis was performed on all datasets provided by the website using GraphPad Prism 8.3.1 (GraphPad Software, San Diego, CA, United States). The types of cancer we analyzed included cancers of the thyroid, lung, colon, head and neck, stomach, liver, pancreas, kidney, urethra, prostate, testis, breast, cervix, endometrium, ovary, glioma, and melanoma. Due to data availability, we focused only on survival data over the first 3 years for glioma and melanoma patients. For all other cancers, we looked at patient survival over 5 years. Of the cancers analyzed, a high level of IQGAP1 mRNA expression was significantly associated with poorer prognosis only in head and neck cancer [51], pancreatic cancer (graph (**A**)), urothelial cancer (graph (**B**)), and melanoma (graph (**C**)).

In general, IQGAP1 is not frequently mutated in cancer, with the possible exception of head and neck cancer [61,62]. Instead, many studies have reported the overexpression of IQGAP1 in cancer tissue samples from patients with colorectal, lung, and breast, as well as head and neck cancers, among others (summarized in Table 1, and reviewed in [11,12]). High levels of expression of IQGAP1 in cancer correlates with poorer prognosis for patients (also summarized in Table 1). Our analyses of RNA-seq data provided by The Cancer Genome Atlas (TCGA) demonstrated that higher levels of IQGAP1 transcripts are significantly correlated with poorer 5-year survival rates in patients with head and neck cancer [51], as well as those with pancreatic cancer (Figure 1), urothelial cancer (Figure 1), and poorer 3-year survival rates in melanoma patients (Figure 1). Interestingly, the subcellular location of IQGAP1 influences its correlation with prognosis: patients with membrane-localized IQGAP1 have lower survival rates than those with cytoplasmic IQGAP1 [63]. In ovarian carcinomas, patients with a diffuse pattern of IQGAP1 expression at the invasion fronts showed a lower overall survival rate than those with a focal pattern [64]. Overall, the level of expression of IQGAP1 and the patterns of IQGAP1 localization within cells show clinical relevance to the prognosis of cancer patients, indicating its importance in cancer development.

In addition to IQGAP1, the IQGAP protein family includes IQGAP2 and IQGAP3, which are both structurally similar to IQGAP1, but are expressed in more limited types of organs [1,12]. The roles of these two proteins, particularly IQGAP3, in carcinogenesis, are less well understood, but the emerging picture is that, while IQGAP3 functions similarly to IQGAP1 as an oncogene, IQGAP2 may serve as a tumor suppressor [12,81,82,83,84,85]. Further studies into how IQGAP2 and IQGAP3 contribute to cancer and how the IQGAP proteins compensate or coordinate with each other will shed more light on IQGAP-mediated carcinogenesis and related therapy designs.

**Figure 2:**
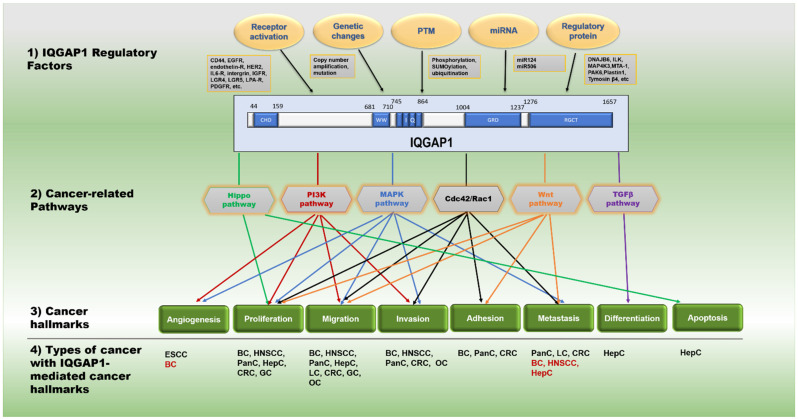
Roles of IQGAP1 in cancer development. (**1**) IQGAP1 regulatory factors: IQGAP1′s activity can be regulated by different cancer development factors, including the genetic changes to the IQGAP1 gene, post-translational modifications on the IQGAP1 protein, activations of signaling receptors, miRNA, or other non-receptor regulatory proteins. Example candidates in each category are summarized in the gray rectangles. (**2**) The IQGAP1 protein: a schematic diagram of human IQGAP1 protein, with each of the five main binding domains highlighted in blue and the numbers above indicating amino acid positions for each domain. (**3**) Cancer-related pathways and cancer hallmarks: list of IQGAP1-mediated pathways and related cancer hallmark events. The pathways and the pathway-regulated hallmarks are color-coded. Dash line indicates a connection between IQGAP1 and this cancer hallmark, but no mechanism is proposed. (**4**) Types of cancer with IQGAP1-mediated cancer hallmarks: BC, breast cancer; CRC, colorectal cancer; ESCC, esophageal squamous cell carcinoma; GC, gastric cancer; HepC, hepatic cancer/liver cancer; HNSCC, head and neck squamous cell carcinoma; LC, lung cancer; OC, ovarian cancer; PanC, pancreatic cancer. Black font indicates that IQGAP1 has been reported to regulate this hallmark through at least one proposed mechanism in this type of cancer; red font indicates that IQGAP1 has been reported to regulate this hallmark in this cancer, but no clear mechanism has been proposed.

Next, we will summarize the current evidence on contributions of IQGAP1 to different types of carcinoma, with a particular emphasis on head and neck cancer, but also including carcinomas of breast, pancreas, liver, colon, gastric, lung, and ovary. These studies together support the hypothesis that IQGAP1 is a crucial regulator of cancer development by scaffolding and facilitating different oncogenic pathways. Figure 2 summarizes the mechanisms from the literature of how IQGAP1, in response to various regulatory factors, contributes to cancer development by mediating multiple cancer-causing pathways.

### 3.1. IQGAP1 in Head and Neck Cancer

Head and neck cancer, mostly head and neck squamous cell carcinoma (HNSCC), arises in the mouth and throat region [86]. HNSCC is the sixth most frequent cancer worldwide [87]. More than 53,000 new cases of HNSCCs and 11,000 associated deaths were estimated in the United States in 2020, with a 5-year survival rate of about 60% [86]. Common etiological factors contributing to HNSCCs include cigarette smoking, alcohol consumption, and human papillomavirus (HPV) infection. [86]. HPV-associated HNSCCs have distinct molecular and clinical features as well as different clinical outcomes from HPV-negative HNSCCs [88,89,90].

Up to 74% of HNSCCs have an activated EGFR/PI3K signaling pathway, with *PIK3CA*, the gene encoding for the catalytic subunit of PI3K, being amplified in > 40% of HNSCCs [91]. IQGAP1 is linked closely to both the PI3K signaling and HNSCC. IQGAP1 binds directly to EGFR and facilitates its activation, and scaffolds components of the downstream PI3K signaling pathway [14,48]. Levels of IQGAP1 are often upregulated in the tumors of HNSCC patients [67,68,92,93,94]. Overexpression of IQGAP1 correlates with poorer HNSCC patient survival [51,67]. Interestingly, a missense mutation in IQGAP1, S459L, was identified in a family predisposed to oral squamous cell carcinoma (OSCC) and this variant was found to be more active than wild-type IQGAP1 in tissue cultures, based upon monitoring levels of MAPK and PI3K signaling [61]. That same study reported somatic mutations, largely missense in nature, in IQGAP1 in sporadic cases of OSCC as well, but the functionality of those mutant IQGAP1 genes was not assessed.

Disrupting IQGAP1′s expression reduced the malignant phenotypes in both laryngeal and esophageal cancer cells [93,94]. IQGAP1 contributes to esophageal cancer by promoting the angiogenesis process through AKT and ERK activation [95]. In nasopharyngeal cancer, metastasis-associated protein 1 (MTA1) regulates IQGAP1 expression and promotes cell proliferation and motility [92]. Inhibition of MTA1 downregulates PI3K signaling in a nasopharyngeal cancer cell line [92]. Targeting the IQGAP1-mediated PI3K signaling using the IQ3 peptide also inhibits human HNSCC cell survival, proliferation, migration, and invasion, indicating the importance of IQGAP1-mediated PI3K signaling in HNSCC cell lines [50,51].

In a recent study, we utilized a genetically engineered mouse strain that is germ-line deficient for IQGAP1 (*Iqgap1^−/−^,* [96]) and found that *Iqgap1^−/−^* mice showed a reduced level of activated PI3K signaling compared to wild-type (*Iqgap1^+/+^*) mice upon EGF stimulation, demonstrating that IQGAP1 is required for efficient EGFR/PI3K signaling in vivo [51]. Utilizing a well-validated mouse model for HNSCC that makes use of a synthetic oral carcinogen (4-nitroquinoline 1-oxide, 4NQO), we discovered that *Iqgap1^−/−^* mice developed significantly lower incidences of cancer, reduced severity (i.e., grade) of disease, and fewer cancer foci per mouse than *Iqgap1^+/+^* mice [51]. IQGAP1 protein levels were upregulated in the HNSCC arising from *Iqgap1^+/+^* mice, consistent with findings in human HNSCC patients [51]. Tumors arising in *Iqgap1^−/−^* mice showed significantly lower levels of PI3K signaling than those in *Iqgap1^+/+^* mice, suggesting that IQGAP1 contributes to HNSCC, at least in part, through PI3K signaling [51]. Meanwhile, the RAS/MAPK pathway, another pathway scaffolded by IQGAP1, was reduced in tumors compared to the adjacent normal epithelium regardless of IQGAP1 status, indicating that the activation of the RAS/MAPK pathway might not be crucial for HNSCCs, at least in this mouse model [51]. In human HNSCC samples, we found that high levels of IQGAP1 correlate positively with high levels of PI3K signaling, further supporting the association between IQGAP1 and PI3K signaling in HNSCCs [51].

About 25% of HNSCCs are associated with HPV, particularly those arising in the oropharynx [97]. The percentage of HPV-associated HNSCC has been rising over the past several decades [98,99,100,101], emphasizing the importance of studying this subtype of HNSCC. PI3K signaling is more frequently altered in HPV-positive HNSCC than HPV-negative HNSCCs [102]. *PIK3CA* is the most frequently mutated gene in HPV-positive HNSCC [102], resulting in the upregulation of PI3K signaling due to gain-of-function mutations in *PIK3CA*. Given the importance of PI3K signaling in HPV-positive HNSCC and the role of IQGAP1 in scaffolding PI3K signaling [48], we hypothesized that IQGAP1 also plays a role in HPV-positive HNSCC. We found that HPV16 E6 upregulated PI3K signaling in normal oral keratinocytes, while E7 only mildly impacted its signaling [103]. Loss of IQGAP1 significantly reduced the PI3K signaling levels in both E6- and E7-expressing keratinocytes [103], indicating HPV16 oncoproteins regulate PI3K signaling in an IQGAP1-dependent manner.

In order to study the role of IQGAP1 in papillomavirus-associated HNSCC in a physiologically relevant scenario, we turned to an infection-based model for HNSCC using the mouse papillomavirus (MmuPV1) that was recently developed in our lab [104]. MmuPV1 can infect the mouse epithelium and cause cancers to arise in different anatomical sites in mice, including the head and neck region [104,105,106,107], which provides opportunities to study the pathogenesis of papillomavirus (PV) in a genetically manipulatable and trackable model organism. MmuPV1 infection upregulates PI3K signaling in keratinocytes [103], which is in line with a previous report showing that HPV16 pseudovirus infection upregulates PI3K signaling in vitro [108]. We also showed that this MmuPV1-induced PI3K signaling was greatly diminished in keratinocytes knocked out for IQGAP1 [103]. Utilizing the MmuPV1 infection-based HNSCC model, we infected the tongues of *Iqgap1^+/+^* (wild-type FVB strain) and *Iqgap1*^−/−^ mice with MmuPV1 (or PBS, mock infection), treated the mice with UVB, which induces systemic immunosuppression that facilitates persistence of infections, and the 4NQO carcinogen to induce HNSCC, and monitored the experimental mice for 6 months. Quantitative PCR (qPCR) analysis of DNA extracted from oral swabs collected at 3 weeks post-infection showed that both MmuPV1-infected *Iqgap1^+/+^* and *Iqgap1*^−/−^ mice carried similar copy numbers of the virus, indicating that IQGAP1 had no measurable effect on incidence of MmuPV1 infection in mice [103]. At 6 months post-infection, MmuPV1-infected *Iqgap1^+/+^* mice developed more severe tumor phenotypes, including tumor incidence, tumor multiplicity and disease severity, that were significantly higher than in MmuPV1-infected *Iqgap1*^−/−^ mice, which had tumor phenotypes very similar to those of mock-infected mice [103]. This result demonstrated that IQGAP1 contributes to MmuPV1-associated HNSCC. The MmuPV1-induced tumors showed features of infection and HPV-associated carcinogenesis [103], consistent with our prior findings [104].

We also tested the role of IQGAP1 in our HPV16-transgenic mouse model (*K14-E6E7* mice), in which the expression of HPV16 E6 and E7 is targeted to the basal layer of the epithelium, under the control of the Keratin 14 promoter (*Krt14*) [109,110]. Consistent with observations in tissue culture, we found that in mice, expression of HPV16 E6 and E7 upregulated PI3K signaling in an IQGAP1-dependent manner [103]. However, IQGAP1 did not significantly impact HPV-associated HNSCC in the 4NQO-induced HNSCC model using these HPV16-transgenic mice [103]. Upon 4NQO oral carcinogen treatment, *Iqgap1*^−/−^*K14-E6E7* mice did not develop significantly less severe cancer phenotypes than the *Iqgap1^+/+^K14-E6E7* mice [103]. Carcinomas arising from both *Iqgap1^+/+^K14-E6E7* and *Iqgap1*^−/−^*K14-E6E7* mice showed similar expression levels and patterns of pERK and pS6, two biomarkers associated with HPV-positive cancer [111,112,113,114], indicating that the loss of IQGAP1 did not reduce the levels of either the RAS-MARK or the PI3K signaling in this model. One explanation for the disparity with the findings in the MmuPV1 infection model compared to the HPV16 transgenic model is that IQGAP1 contributes more to earlier stages of PV-associated carcinogenesis, which can be captured by the MmuPV1-infection model, but not the HPV16-transgenic mouse model. More in-depth investigations are needed to explain the discrepancy in the observations made with the two different models we used, which could help us better understand how HPV induces HNSCC and how IQGAP1 plays a part in this process, and hopefully lead to new drug targets for HPV-associated HNSCC.

RAC1/CDC42 signaling may be another way for IQGAP1 to contribute to HPV associated HNSCC [115,116,117]. HNSCC cell lines often carry hyperactivated RAC1 [118]. RAC1 can be activated by HPV18 E6, while HPV16 E6 activates CDC42 instead [116,117]. RAC1 plays an essential role in HPV8-associated tumorigenesis in mice [115]. RAC1 inhibition suppresses HPV8-associated papilloma formation in mice, while RAC1 activation facilitates papillomatosis [115]. Given that IQGAP1 can bind to both RAC1 and CDC42 and regulate their activation [4,5,8,24], IQGAP1 may potentially be involved in HPV-associated tumorigenesis through RAC1/CDC42 signaling.

Interestingly, the JNK pathway was recently implicated in the context of HPV-associated cervical cancer [119]. HPV16 and HPV18 E6 induce JNK activation via the PDZ-binding domains [119]. The JNK pathway contributes to HPV-associated cervical cancer cell proliferation, anchorage-independent growth, migration, invasion and EMT [119] Inhibiting the JNK pathway reduced EGFR’s expression levels in HPV-associated cervical cancer cell lines [119]. IQGAP1 is required for efficient HPV16 E6-induced PI3K signaling, which is downstream of EGFR [103]. IQGAP1 is also linked to JNK activation [23]. Since JNK signaling is also implicated in oral cancers [120], it is possible that IQGAP1 contributes to HPV-associated HNSCC through the JNK pathway. More research is needed to understand the role of IQGAP1 in HNSCC in the context of the JNK pathway.

### 3.2. IQGAP1 in Breast Cancer

IQGAP1 was first discovered to promote tumorigenesis in breast cancer [38]. Inhibiting IQGAP1 reduced anchorage-independent growth, migration, and invasion of MCF7 breast cancer cells in vitro, and tumor growth and vascularization of MCF7 xenografts in vivo [8,38]. Subsequent studies demonstrated that IQGAP1 contributes to breast cancer through multiple pathways. IQGAP1 can bind directly to the estrogen receptor (ER), a major contributor to breast cancer progression, and promote estrogen-dependent transcription and cell proliferation [121]. IQGAP1 also binds directly to the HER2 receptor, which is overexpressed in 20–25% of breast cancers, and mediates its downstream signaling [21]. Knocking down IQGAP1 inhibits growth of HER2-positive breast cancer cells and increases their response to trastuzumab, an antibody-based drug that inhibits the activity of the HER2 receptor [21]. IQGAP1 can also promote breast cancer cell proliferation and migration through the RAS-MAPK pathway and CDC42/RAC1 pathways [38].

In ER-negative breast cancer cell lines, IQGAP1 promotes EGFR activation upon stimulation with the metastasis-related KISS1 peptide and induces invasion and EMT, a critical step in metastasis [39]. IQGAP1 is also responsible for lysophosphatidic acid (LPA)-induced cell migration and invasion [40]. Targeting IQGAP1 does not impact the primary ER-negative tumor growth but reduces its spontaneous metastasis to both the lung and liver [122]. MicroRNAs (miRNAs), important regulators of a wide range of cellular processes, also impact breast cancer development through IQGAP1. miR-506, which is often downregulated in breast cancer, was reported to target the 3′UTR of IQGAP1 and impact cell proliferation, invasion, and adhesion, possibly through the RAS-MAPK pathway [123].

IQGAP1-mediated PI3K signaling is also critical for breast cancer survival. Suppressing IQGAP1-mediated PI3K signaling using a cell-permeable peptide, IQ3, which contains the PI3K binding motif on IQGAP1, inhibited the survival of human breast cancer cells, regardless of the status of ER expression or the presence/absence of gain-of-function mutations in the *PIK3CA* gene, which encodes for the catalytic subunit of PI3K [48].

### 3.3. IQGAP1 in Pancreatic Cancer

Up to 90% of pancreatic cancers carry activating mutations in *KRAS* [124]. Mutated *KRAS* increases phosphorylation of ERK, indicating that RAS-MAPK signaling contributes significantly to pancreatic cancer progression [124]. Given that IQGAP1 can scaffold the RAS-MAPK signaling pathway, it is not surprising that IQGAP1 has an essential role in pancreatic cancer. Knocking down IQGAP1 using shRNA reduced the abilities of pancreatic cancer cells to proliferate and migrate and reduced the incidence of tumor formation and liver metastases in pancreatic cancer xenograft models in vivo [125,126]. The addition of the cell-permeable WW peptide, which contains IQGAP1′s binding motif to the ERK protein, and therefore specifically blocks IQGAP1-mediated ERK activation, decreased tumor growth both in xenografts models and in a transgenic mouse model that spontaneously develops lethal pancreatic cancer, and extending the life span of the transgenic mice [22,125]. Fructose-1,6-bisphosphatase (FBP1), a gluconeogenesis enzyme that is often downregulated in cancer, can also directly bind to the WW domain of IQGAP1 and therefore inhibit IQGAP1-mediated ERK activation [127]. An FBP1-derived peptide decreased pancreatic cancer cell growth and migration both in vitro and in vivo [127]. Gemcitabine is one of the major treatment options for pancreatic cancer, the use of which is hindered by both intrinsic and acquired chemoresistance [128]. Gemcitabine-resistant pancreatic ductal adenocarcinoma (PDAC) cells showed high levels of pERK [129]. FBP1-derived peptide helped sensitize PDAC cells to Gemcitabine treatment [127], raising the possibility that IQGAP1 might play a role in resistance to Gemcitabine.

IQGAP1 also promotes pancreatic cancer through other pathways. Proinflammatory cytokine Interleukin-6 (IL6) is often upregulated in pancreatic cancer and is required for cancer progression in vivo [33]. IL6 activates the JAK-STAT3 pathway, leading to STAT3 interacting with IQGAP1 and activating CDC42 through IQGAP1, thereby inducing CDC42-mediated cell invasion [33]. The knockdown of IQGAP1 reduced cell proliferation and migration in pancreatic cancer cells in a CDC42/RAC1-dependent manner [126]. Active RAC1 can disrupt the interaction between IQGAP1, E-cadherin and β-catenin and reduce cell–cell adhesion in pancreatic cancer cells [36]. Additionally, IQGAP1 promotes EMT in pancreatic cells through canonical Wnt signaling by interacting with DVL2 and β-catenin [70]. Together, the literature suggests that IQGAP1 could be a valuable therapeutic target in treating patients with pancreatic cancer, potentially even those patients with cancers that display intrinsic/acquired drug resistance.

### 3.4. IQGAP1 in Liver Cancer

Both IQGAP1 and the related protein IQGAP2 significantly impact the development of liver cancer. IQGAP2 has a similar protein structure as IQGAP1, and is mostly expressed in the liver, kidney, and platelets [130]. However, unlike IQGAP1, IQGAP2 serves as a tumor suppressor in the liver [130]. The role of IQGAP1 and IQGAP2 in liver cancer has been reviewed previously [130]. Briefly, levels of IQGAP1 are commonly increased while IQGAP2 levels are decreased in liver cancer [46,72,130,131]. Patients with high levels of IQGAP1 but low levels of IQGAP2 had the worst prognosis, while those with low levels of IQGAP1 but high levels of IQGAP2 had the best outcomes [72]. The loss of IQGAP2 in mice spontaneously leads to liver cancer development, which is also IQGAP1-dependent [132].

Inhibiting IQGAP1 expression reduced cell proliferation, migration, and EMT, and increased apoptosis in liver cancer cells, indicating the importance of IQGAP1 in liver cancer cell maintenance [71,133,134,135]. Knocking down IQGAP1 resulted in lower levels of RAS proteins [134]. IQGAP1 also contributes to liver cancer through Wnt signaling, in which IQGAP1 interacts directly with β-catenin and regulates β-catenin-dependent transcription, impacting cell proliferation and migration [136]. However, IQGAP1 alone is not sufficient to cause liver cancer [55]. Interestingly, the absence of IQGAP1 modestly increases tumorigenesis in a carcinogen-induced liver cancer model, which may be due to increased levels of MET receptor kinase signaling [55]. In the presence of active Wnt and MET signaling, overexpression of IQGAP1 promotes the formation of highly aggressive liver cancer [55]. This indicates that, though IQGAP1 is not required for liver carcinogenesis, it can still promote cancer progression in some settings.

The Hippo pathway controls the organ size and regeneration in the liver [137]. Bile acid is a common factor in liver cancer and upregulates the Hippo pathway both in vitro and in vivo in an IQGAP1-dependent manner [53,138]. In an animal model, bile acid treatment induces IQGAP1 expression [138], which reduces YAP phosphorylation and therefore activates YAP [53,138]. In the absence of IQGAP1, bile acid failed to promote cell proliferation due to the reduced levels of IQGAP1-activated YAP [138]. In liver cancer cells, the bile acid-induced overexpression of IQGAP1 decreased YAP1′s phosphorylation, which inhibits both YAP1-p73 regulated transcription of apoptosis-related genes and pro-growth YAP1-TEAD transcription [53]. More studies are needed to determine whether IQGAP1 contributes to liver cancer via the Hippo pathway.

Interestingly, IQGAP1 seems to have a different role when expressed in the tumor microenvironment. In hepatic stellate cells (HSCs), the resident liver pericytes, IQGAP1 suppresses TGF-β-mediated signaling and prevents myofibroblast differentiation, which is a key step in promoting tumorigenesis [57]. *Iqgap1^−/−^* mice showed higher levels of liver metastasis than *Iqgap1^+/+^* mice when implanted with mouse colon and lung cancer cells. When co-implanted with colon cancer cells into nude mice, HSCs with reduced levels of IQGAP1 promoted tumor growth, and led to increased levels of TGF-β receptor and myofibroblast activation in the resulting tumors [57]. Further studies investigating the different roles of IQGAP1 in epithelial carcinoma cells vs. in stromal cells could lend additional insight into how IQGAP1 contributes to primary liver cancer and to liver metastases.

### 3.5. IQGAP1 in Colorectal Cancer

Dysregulation of Wnt signaling is essential for colorectal cancer (CRCs) development, in which close to 100% of CRCs carry alterations in Wnt signaling [41]. IQGAP1 interacts with multiple components in the canonical Wnt pathway and increases β-catenin-dependent responses [42,44]. PAK6, which is overexpressed in cancer and phosphorylates β-catenin, might disrupt CRC cell–cell adhesion through the PAK6/IQGAP1/E-cadherin complex at the cell junction [139]. However, another study reported that LGR5, which potentiates Wnt signaling upon R-spondin (RSPO) binding, interacts directly with IQGAP1, reduces IQGAP1 phosphorylation, enhances the IQGAP1-RAC1-actin association, and eventually increases cell–cell adhesion of CRC cell lines [35]. These studies together indicate that IQGAP1 has different roles in CRC cell–cell adhesion, depending on the interacting complex. Both Thymosin β4 and Plastin 1 were reported to regulate CRC cell migration through IQGAP1-Rac [93,94]. Knockdown of IQGAP1 reduced cell invasion by CRC cells [74]. DNAJB6, a heat shock protein that is overexpressed in CRC, also promoted CRC invasion and metastasis through IQGAP1-ERK signaling [140]. Interestingly, PTM of IQGAP1 also plays a role in CRC. IQGAP1 has increased levels of SUMOylation in CRC [141]. The SUMOylation of IQGAP1 increased CRC cell growth, migration, and tumorigenesis, possibly because SUMOylation stabilizes IQGAP1 by reducing its ubiquitination [141]. Additionally, miR-124, a microRNA decreased in CRC, targets IQGAP1, and reduces CRC cell proliferation and colony-forming ability, possibly through a reduced activity of ERK and β-catenin [142].

### 3.6. IQGAP1 in Lung Cancer

Several studies argue that IQGAP1 plays a role in lung cancer by facilitating Wnt signaling. Colocalization of IQGAP1 and DVL in the cytoplasm and nucleus positively correlates with poorer prognosis in patients with non-small cell lung cancer (NSCLC), the most common type of lung cancer, and increased lymph nodal metastasis [143]. Overexpression of IQGAP1 in bronchial epithelial cells increased nuclear β-catenin, activated TCF, and upregulated levels of c-MYC and cyclin-D1, two oncoproteins that are under Wnt control [144]. In *KEAP1*-deficient lung cancer, RSPO3, the ligand that activates Wnt signaling, is upregulated due to the Keap1 deficiency and, along with its receptor LGR4, mediates cell migration and metastasis in an IQGAP1-dependent manner [145]. Other than Wnt signaling, MAP4K3, a member of the mitogen-activated protein kinase kinase kinase kinase (MAPK4) family, regulates lung cancer metastasis through IQGAP1 [146]. MAP4K3 binds directly to and phosphorylates IQGAP1, which enhances CDC42 activation and, therefore, cell migration [146]. The interaction of MAP4K3 and IQGAP1 was associated with poorer prognosis in lung cancer, indicating the importance of IQGAP1-mediated signaling.

IQGAP1 is also reported to influence stromal cells of the lung. Recent transcriptome analysis demonstrated that IQGAP1 likely regulates cell proliferation, cell adhesion, and cell migration in pulmonary microvascular endothelial cells, as well as the production of cytokines and inflammatory factors [147]. Upon EGFR inhibitor-targeted therapy, a standard first-line treatment for NSCLC, IQGAP1 is significantly increased in microvascular endothelial cells, which decreases cell–cell adhesion, increases endothelial cell permeability, and eventually contributes to treatment-induced vascular adverse events, such as purpuric drug eruptions [148]. A better understanding of the different effects of IQGAP1 on epithelial vs. stromal cells could help identify better treatments for lung cancer patients.

### 3.7. IQGAP1 in Gastric Cancer

The role of IQGAP1 in gastric cancer is not clear. The IQGAP1 gene is amplified in gastric cancer cell lines [149], and levels of IQGAP1 protein are increased in gastric cancers, [76,77,149]. Membrane-localized IQGAP1 is associated with higher grades of gastric cancer, possibly due to reduced cell–cell adhesion [76]. RhoC, a Rho GTPase overexpressed in gastric cancer, binds to IQGAP1 and regulates cell proliferation and cell migration in an IQGAP1-dependent manner [150,151]. Another study suggested that integrin-like kinase (ILK) activates NF-κB through IQGAP1-mediated RAS-MAPK signaling and regulates cell migration and proliferation in gastric cancer cells [152]. These studies together would support the hypothesis that IQGAP1 acts as an oncogene.

On the other hand, some evidence indicates that IQGAP1 can act as a tumor suppressor in gastric cancer. The loss of IQGAP1 induces gastric hyperplasia in *Iqgap1*^−/−^ mice [96], consistent with IQGAP1 acting as a tumor suppressor. In another study focusing on *Helicobacter(H.) pylori* infection, which can cause gastric cancer, mice lacking IQGAP1 tend to develop a higher incidence of gastrointestinal neoplasia upon infection with *H. pylori* [153]. More studies are needed to determine whether this is due to the different role of IQGAP1 in the gut between different species (humans vs. mice), or if it is due to the complexity of IQGAP1-mediated signaling in which IQGAP1 regulates different signaling pathways at different concentrations, as has been suggested by others [154].

IQGAP1 was also linked to cancer drug resistance. In patients with HER2-positive advanced gastric cancers, the efficacy of HER1-targeted trastuzumab treatment is limited by acquired drug resistance against trastuzumab [155]. Increased levels of IQGAP1 were observed in gastric cancer cell lines with acquired trastuzumab resistance. Inhibiting IQGAP1 re-sensitized these cells to trastuzumab treatment in vitro, indicating a role of IQGAP1 in mediating trastuzumab resistance [155]. In patients treated with taxane-containing chemotherapy, levels of asparaginyl endopeptidase (AEP) increase and play an important role in acquired drug resistance [156]. AEP interacts with IQGAP1 and possibly contributes to drug resistance through IQGAP1-mediated RAS-MAPK signaling [156].

### 3.8. IQGAP1 in Ovarian Cancer

Ovarian cancer remains the leading cause of death amongst gynecological cancers, with minimal mortality improvement over the past decade [157,158]. Estrogen is highly implicated in ovarian cancer development [157]. Since IQGAP1 can bind to ERα and increase ERα-dependent transcription [121], it is logical to hypothesize that IQGAP1 plays a role in ovarian cancer. Current literature investigating IQGAP1′s role in ovarian cancer, though very limited, suggest that IQGAP1 promotes ovarian cancer by upregulating cell migration, invasion, and metastasis. Inhibiting IQGAP1 in ovarian cancer cells did not impact proliferation but reduced cell migration and invasion [159]. Upon activation of CD44, which correlates with ovarian tumor cell invasive behavior, a signaling complex of CD44-IQGAP1-RAC1 formed to regulate actin activation, contributing to tumor cell migration [160]. This signaling complex also binds and phosphorylates ERK2, therefore activating downstream signaling including the transcription activities controlled by Elk1 and ERα that contribute to tumor progression [160]. In spheroid cultures, levels of IQGAP1 increased during the differentiation of ovarian cancer stem cell-like cells (CSC-LCs), and enhanced the invasion properties of CSC-LCs [161]. IQGAP1 is also involved in ovarian cancer metastasis. Upon signaling through integrins or endothelin-1, a key regulator of ovarian tumor progression, IQGAP1 forms a signaling complex with RacGAP1, which inactivates Rac but activates RhoA, thereby promoting the formation of invadopodia [158,162]. Inhibiting endothelin-1 receptor binding reduced ovarian cancer cell metastasis in vivo and impaired the expression of IQGAP1 and other invadopodia effectors in the metastasized tumors [162]. Further investigation into how IQGAP1 impacts ovarian cancer and strategies targeting IQGAP1-mediated signaling could lead to new, effective treatments for ovarian cancer patients.

## 4. Links between IQGAP1 and Immune Cell Activities

Emerging evidence has implicated IQGAP1 in regulating immune cell functions [1]. Actin filaments and microtubules are important for controlling immune cell polarity, adhesion, migration, infiltration, endocytosis and phagocytosis [163,164,165,166]. IQGAP1 can influence these immune cell activities by regulating the cell cytoskeleton [167,168,169,170,171,172,173,174,175]. IQGAP1 regulates phagocytosis by macrophages and increases macrophage infiltration [173,174,176]. IQGAP1 also promotes natural killer (NK) cell motility [172], and is required for microtubule organizing centers (MTOC) and granule polarization in NK cells during synapse maturation [175]; IQGAP1 binds to CXCR2 and thereby may play a role in regulating neutrophil migration [171]; IQGAP1 also participates in B-cell receptor-induced MTOC polarization at the immune synapse between B cell and antigen-presenting cells [167]. In T cells, however, IQGAP1 negatively regulates the accumulation and the movement velocity of F-actin at the immune synapse [168], an essential cytoskeleton-associated process for proper immune synapse function [177].

Tumor-associated macrophages (TAMs) promote cancer development [178]. Macrophages isolated from the bone marrow of *Iqgap1^−/−^* mice show decreased levels of migration and adhesion, consistent with the observation that *Iqgap1^−/−^* mice had lower levels of macrophage infiltration in tissues [176]. Moreover, the overexpression of IQGAP1 correlates with a higher number of TAMs in head and neck cancers [67], indicating the possibility that IQGAP1 could be contributing to carcinogenesis by promoting infiltration of TAMs.

NK cells are effector lymphocytes that target tumor cells but with low capabilities to infiltrate the tumor microenvironment [179]. IQGAP1 regulates the cytotoxic activity of an NK-like cell line [175] and mediates the MAPK pathway in NK cells [169]. IQGAP1 also activates NK-cells and their anti-tumor responses in a mouse model [172]. However, the exact mechanisms of how IQGAP1 influences NK cells during cancer progression is still largely unknown.

In contrast, IQGAP1 functions as a negative regulator for T cells. The loss of IQGAP1 upregulated T-cell receptor-mediated signaling and related cytokine production [168]. Primary and naïve CD8+ T cells isolated from *Iqgap1^−/−^* mice displayed increased cytokine production upon stimulation [180]. Interestingly, a new study established a novel link between IQGAP1 and OX40, a T cell co-stimulatory receptor, in which IQGAP1 binds to OX40 and constrains its signaling activity [181]. CD4+ T cells derived from *Iqgap1^−/−^* mice showed increased proliferation and cytokine production levels compared to those from the wild-type mice upon OX40 stimulation [181]. When experimental autoimmune disease was induced, *Iqgap1^−/−^* mice showed higher levels of immune cell infiltration and antigen-specific effector T-cell responses [181]. This is intriguing because OX40 has been proposed to be a co-target in immunotherapy, in which anti-OX40 antibodies prolong the survival of effector T cells and impair the immune-suppressing properties of regulatory T cells [182]. Therefore, it is possible that targeting IQGAP1 could increase responses to immunotherapy by upregulating OX40 signaling.

## 5. Conclusions

IQGAP1 promotes cancer progression by scaffolding different signaling complexes. In the past decade, evidence has emerged on new roles played by IQGAP1 in signal transduction in carcinogenesis. Given the broad nature of IQGAP1-interacting partners, it is essential to study the dynamics between IQGAP1-mediated pathways in the context of cancer. With a better understanding of the role of IQGAP1 in cancer and the underlying mechanism, therapies targeting IQGAP1 and its related signaling will potentially be beneficial, especially since many cancers overexpress IQGAP1 and/or depend upon IQGAP1-mediated signaling. Additional studies need to be pursued regarding the other two IQGAP proteins, IQGAP2 and IQGAP3, which may have either opposing or complementary activities to IQGAP1 depending on context, and thereby may affect outcomes of any IQGAP1-targeted therapies. This is particularly true for IQGAP3, which scaffolds similar signaling pathways as IQGAP1, and therefore might compensate for IQGAP1′s functions under conditions in which IQGAP1 is targeted.

The emerging insights on IQGAP1′s role in immune regulation are particularly intriguing. The new insights that have emerged about IQGAP1′s role in tumor-associated immune cells, especially TAMs and T cells, might intensify the efficacy of direct cancer-killing effects and enhance beneficial immune response at the same time. Of particular note, IQGAP1-mediated OX40 signaling may improve responsiveness to immune checkpoint blockade immunotherapy, such as the anti-PD1/PDL1 treatment.

## Figures and Tables

**Table 1:** List of cancer types with overexpressed IQGAP1.

Cancer Type	Method	Target	Comparison	Prognostic *	References
Breast	IB	protein	cancer vs. normal	yes	[65,66]
	IHC	protein	cancer vs. normal		
	RT-PCR	mRNA	cancer vs. normal		
	IHC	protein	high-grade vs. low-grade cancer		
Head & neck	MS	protein	cancer vs. normal	yes	[51,67,68]
	IHC	protein	cancer vs. normal		
Pancreas	IHC	protein	cancer vs. normal	yes	[69,70]
	IB	protein	cancer vs. normal		
	IHC	protein	high-grade vs. low-grade cancer		
Liver	IHC	protein	cancer vs. normal	yes	[46,71,72]
	IB	protein	cancer vs. normal		
Colorectal	Array	gene	cancer vs. normal	yes	[73,74,75]
	IHC	protein	cancer vs. normal		
Gastric	IB	protein	cancer vs. normal	no	[76,77]
	IHC	protein	high-grade vs. low-grade cancer		
Lung	RT-PCR	mRNA	cancer vs. normal	yes	[63,78,79,80]
	IHC	protein	high-grade vs. low-grade cancer		
Ovary	IHC	protein	adenocarcinoma vs. adenoma	yes	[64]

This table is an adaptation from [12]. * “Yes” indicates that, based upon cited studies, high expression of IQGAP1 correlates with poorer prognosis for this type of cancer. IB: immunoblotting; IHC: immunohistochemistry; MS: mass spectrometry; RT-PCR: reverse transcription-polymerase chain reaction.

## Data Availability

Not applicable.
